# ‘It reshaped how I will do research’: A qualitative exploration of team members’ experiences with youth and family engagement in a randomized controlled trial

**DOI:** 10.1111/hex.13206

**Published:** 2021-02-15

**Authors:** Natasha Y. Sheikhan, Lisa D. Hawke, Kristin Cleverley, Karleigh Darnay, Lynn Courey, Peter Szatmari, Amy Cheung, Joanna Henderson

**Affiliations:** ^1^ Centre for Addiction and Mental Health Toronto Ontario Canada; ^2^ Department of Psychiatry University of Toronto Toronto Ontario Canada; ^3^ Lawrence S. Bloomberg Faculty of Nursing University of Toronto Toronto Canada; ^4^ Sashbear Foundation Toronto Ontario Canada; ^5^ Sunnybrook Health Sciences Centre Toronto Ontario Canada

**Keywords:** adolescent, caregivers, family engagement, family relations, mental health, qualitative research, youth engagement

## Abstract

**Background:**

Engaging youth and family members as active partners in research and service design offers great promise in improving projects. In youth mental health, recent research has highlighted the value of youth and family engagement. However, research on the experience and impacts of engagement is sparse.

**Objective:**

This study explores the project team's experience of youth and family engagement in the design and development of the YouthCan IMPACT randomized controlled trial and clinical service pathway design.

**Design:**

Qualitative data collected using semi‐structured interviews and a focus group as part of the YouthCan IMPACT clinical trial were analysed to understand the impacts of engagement. Twenty‐eight team members were interviewed, including youth and family members. A qualitative content analysis was conducted, with a member checking process.

**Results:**

Team members reported facilitators, barriers and impacts of youth and family engagement. Facilitators included a safe environment and strong procedures conducive to inclusion in co‐design. Barriers included logistical, structural and institutional constraints. Overall, team members found youth and family engagement to be valuable and to positively impact the research and service design process.

**Discussion and Conclusions:**

Youth and family engagement played a critical role in research and clinical service pathway design. The team found that their involvement improved the quality of the research and service pathway through sustained and multifaceted engagement. Facilitators and barriers to engagement may serve to guide future engagement initiatives. Future research should evaluate the long‐term impact of early engagement and further focus on family engagement.

**Patient/Public Contribution:**

Youth and family members were engaged in the data analysis and interpretation process.

## 1 INTRODUCTION

There is a growing awareness of the need to understand youth and family perspectives in research and service design.^1^, ^2^, ^3^ In the health sphere, the last decade has demonstrated a shift towards engaging people with lived experience in research and service development. While this is true across health disciplines, youth mental health research has also seen an increase in engagement.^4^

Youth have unique needs and face specific barriers in mental health and substance use service access and treatment. In Canada, the response thus far to youth mental health and substance use challenges has been largely considered inadequate, where youth face barriers such as stigma and significant delays in accessing care.^2^, ^5^, ^6^ To better address service access barriers, youth‐serving agencies in Canada and worldwide are collaborating with youth and family members in the design and delivery of services, while some research groups are also engaging youth and family members in research evaluating such services.^4^, ^7^, ^8^

Youth and family engagement in research and service design and delivery involves incorporating a broad range of youth and family perspectives throughout the initiative to ensure full partnership.^7^, ^9^ Engagement shifts service users from being passive participants to active partners in research and service design, hopefully resulting in mutual benefits. Engaged service users have reported empowerment and skill‐building,^10^ while researchers have reported enhanced research innovation.^11^ Given that the majority of mental health and substance use problems develop before the age of 24,^12^ and considering the value of service user engagement,^13^, ^14^ integrating youth perspectives is critical to improving the quality of care. Since family members can have an important role in youth's service‐seeking decisions, behaviours and outcomes, they should also be engaged in youth mental health service and research design.^15^

Recent research highlights the importance of, and ways to incorporate, youth engagement in research and service design.^16^, ^17^, ^18^ For instance, a scoping review^16^ highlights the importance of incorporating youth voices throughout service design and delivery as a core value in youth‐friendly services. Although a small body of research has begun to illustrate methods of engaging service users as active partners in research,^14^, ^19^ there is a dearth of research on the impacts of youth and family engagement. There is a particular paucity of literature regarding family engagement. Furthermore, youth and family perspectives are still rarely integrated throughout service planning, development and research. Without robust research demonstrating the value of engagement, clinical programs and research project teams may overlook youth and family engagement and thereby miss the benefits that engagement can provide.^2^, ^20^

Integrated youth service hubs (IYSHs) are a growing model of youth care that aims to increase service access and reduce system fragmentation through a ‘one‐stop‐shop’ approach.^4^, ^8^ They do so through a variety of core principles, including rapid access, evidence‐informed approaches and a concerted focus on youth and family engagement in service design and evaluation.^4^, ^8^, ^9^ Examples of emerging IYSH models around the world include ACCESS Open Minds across Canada,^21^ YouthCan IMPACT in Toronto, Ontario, Canada,^9^ Youth Wellness Hubs Ontario in Ontario, Canada,^22^ Foundry in British Columbia,^23^ Jigsaw in Ireland^24^ and Headspace in Australia.^25^ Studies evaluating IYSHs illustrate benefits such as a reduction in distress, self‐reported mental health improvements and high levels of satisfaction.^8^ However, experts have identified a need for more rigorous research on engagement.^4^, ^8^, ^18^, ^26^, ^27^

**Figure 1:**
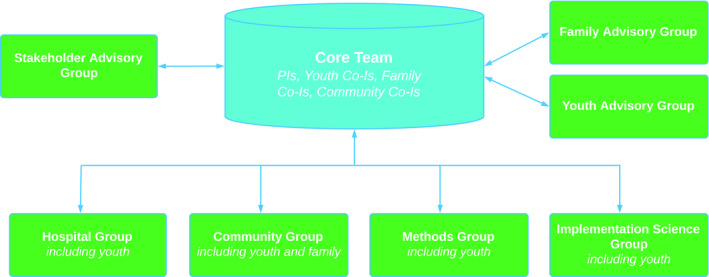
YouthCan IMPACT Governance Model. CIs, co‐investigators; PIs, principal investigators. This figure is reproduced under a Creative Commons license from Henderson et al.^29^

The YouthCan IMPACT project in Toronto, Canada, consists of the development and evaluation of an IYSH model incorporating youth‐friendly components, such as rapid service access, evidence‐based interventions and system navigation.^28^ To reflect youth and family expertise at all levels of service and research design and ensure effective partnership and collaboration, the YouthCan IMPACT team created a governance model in which youth and family members with lived and living expertise are part of the core decision‐making body and working groups (Figure 1).^9^ Youth and family expert advisory groups were also created, to integrate feedback from a broader group of youth and family members.

A recent study evaluated the start‐up process of YouthCan IMPACT, highlighting barriers and facilitators to implementation.^29^ However, as limited evidence exists regarding the impact of youth engagement in service design—and even less so on family engagement—the impact of engagement in the project remains unclear.^4^

In the McCain Model of Youth Engagement,^7^ which was co‐developed by youth and researchers, youth can be engaged in a variety of ways. These methods range from low to high commitment, but all aim to bring a broad range of skills to a project and promote youth decision‐making power.^7^ The model embodies core principles of authentic decision making (*e.g.,* active participation in research decision making), flexibility (*e.g.,* around their role), informal and formal mentorship (*e.g.,* around research or policy) and reciprocal learning, where both youth and researchers are considered experts. The McCain Model was implemented in YouthCan IMPACT to facilitate authentic youth engagement. The general principles of the model were also extended to guide family engagement.

This study explores the team's experience of youth and family engagement in the design and development of the YouthCan IMPACT randomized controlled trial and clinical service pathway.

## 2 METHODS

### 2.1 The YouthCan IMPACT project

The YouthCan IMPACT project is a community‐based service development project and clinical trial that created an integrated collaborative care team model of service delivery in Toronto, Ontario, Canada.^28^ The project began in 2015 as a patient‐oriented randomized controlled trial at the Centre for Addiction and Mental Health (CAMH). The project operates out of the Margaret and Wallace McCain Centre for Child, Youth and Family Mental Health (McCain Centre), the centre responsible for developing the McCain Model of Youth Engagement. The McCain Model emerged in parallel with the YouthCan IMPACT project and therefore was implemented to engage youth throughout the project. Using the McCain Model, youth and family members were involved in all phases of the project, including research design, service pathway development, analysis, evaluation and implementation.

### 2.2 Study data and setting

This study is a secondary of qualitative interview data from the YouthCan IMPACT formative evaluation,^29^ to understand the impact of youth and family engagement. The formative evaluation was conducted during the start‐up phase of the project to understand the team's experience of the service development, research design and implementation processes. The study received ethical approval from the CAMH Research Ethics Board.

### 2.3 Sample

The sample consisted of 28 YouthCan IMPACT project team members who consented to be interviewed. Team members were principal investigators, project coordinators, managers, clinicians, community leads and youth and family leads. Study participants in the randomized controlled trial were not interviewed. The interviewer and lead analyst were not interviewed in the study and were not members of the core research team. Participants were recruited through direct email or in‐person invitations. Written informed consent was obtained from participants.

### 2.4 Data collection

Participants took part in semi‐structured interviews and a focus group during the start‐up process of YouthCan IMPACT. All interviews were carried out by a research staff member, a medical student at the time. Interviews were conducted face to face and via telephone. Twenty‐two interviews were conducted from August 2016 to April 2017, ranging from approximately 25 minutes to 105 minutes (average 53 minutes). The 22 interviews consisted of 17 semi‐structured interviews with one participant, four semi‐structured interviews with two participants and one focus group with four participants. Interview questions were based on the Consolidated Framework for Implementation Research (CFIR),^30^ which specifies domains related to implementation success. The CFIR structure was the focus of the companion manuscript^29^ and was not adapted specifically to query about the engagement process. Questions were further developed through a document review (*e.g.* meeting minutes). Interviews were recorded, transcribed and entered into NVivo 12. Tracking logs of all scheduled team meetings were reviewed to quantify youth and family attendance.

### 2.5 Data analysis

Data were analysed using content analysis through a deductive approach.^31^ All transcripts were analysed and coded by one member of the research team (NYS). A second coder analysed 20% of the transcripts. Initial codes were formed though open coding, and then, categories were created from the raw data. The codes and categories were then discussed with another researcher (LDH) to review diverging opinions regarding the categorization of data.^32^ While both the interviewer and lead data analyst were supported by a team member who was also a study participant, data were blinded from the analyst and supporter to reduce bias. Following the organization phase, the codes and categories were shown to youth and family consultants within the YouthCan IMPACT engagement initiative for member checking.^33^ This ensured that the interpretation reflected youth and family perspectives. The initial codes were revised accordingly. Results were then described by the content of the categories using the deductive approach.

## 3 RESULTS

For participant characteristics, see Table 1. Participants represented a variety of roles and included youth and family members. The majority were female. The frequency of meeting attendance by youth and family members is shown in Table 2. From June 2015 to August 2017, youth were present in 203 meetings, and family representatives were present in 59 meetings, including the core leadership team and working groups that developed the clinical pathway and research methodology. As recommended by our youth engagement team, the categories from the content analysis are organized in terms of (1) general findings, (2) youth‐specific findings and (3) family‐specific findings. Facilitators and barriers to youth and family engagement are shown in Tables 3 and 4. Impacts of engagement are shown in Table 5.

**Table 1:** Characteristics of study participants

	Participants N = 28
*Gender*	
Male	3
Female	25
*Role*	
Community clinical or service partner	8
Principle or co‐investigator	5
Research manager or coordinator	2
Community manager	5
Youth and family	3
Community lead	3
Other collaborative staff	2

**Table 2:** Number of project team meetings attended by youth and family members from project start‐up phase in 2015 to August 2017

	Group type				Total
	Core	Methods	Community	Implementation science	
Youth	117	37	46	3	203
Family	41	0	18	0	59
Total	158	37	64	3	262

Note

A total of 7 youth and 2 family members were engaged during this time period. In addition to these meetings, multiple youth advisory and family advisory meetings were held.

**Table 3:** Facilitators to engagement in the YouthCan IMPACT project

Facilitators	Youth	Family
*Environmental facilitators*		
Youth and family‐friendly language	x	x
Efficient decision making	x	x
Inclusive environment	x	x
Non‐judgemental environment	x	x
Safe space provided by research team	x	x
Safe space enhanced by youth and family presence	x	x
Diversity among voices	x	
Previous team relationships	x	x
*Procedural facilitators*		
Treated as equals	x	x
Co‐creation process	x	x
Pre‐ and de‐briefs	x	
Relationship‐building activities	x	x

**Table 4:** Barriers to engagement in the YouthCan IMPACT project

Barriers	Youth	Family
*Logistical barriers*		
Time	x	x
Rushed process	x	x
Scheduling	x	
*Structural and institutional barriers*		
Newness of engagement in the discipline	x	x
Limited funding	x	x
*Engagement structure and process*		
Less supported		x
Need for stronger supports in early stages	x	
Resistance to certain changes	x	
Navigating different backgrounds and perspectives	x	x
Lack of youth with no experience in field	x	
Lack of continuity in roles and growing out of roles	x	
Learning curve	x	x

**Table 5:** Impacts of engagement in the YouthCan IMPACT project

*Perceived value*		
Informing various services, ideas and outcomes	x	x
Considered valuable	x	x
*Meaningful experience*	x	x
Substantial impact on the success of the project	x	x
*Positive impacts on research*		
Enhanced researchers' commitment to engagement	x	x
Improved research decision making	x	
Good relationship with team	x	x
Improved selection of research measures	x	
*Contributions to project and service design*		
Contributed ideas to the project	x	x
More efficient decision making about services and research	x	x
Improved service design	x	x
Services, ideas and outcomes	x	x

### 3.1 General findings across youth and family engagement

#### 3.1.1 Importance of engagement

Team members who discussed engagement in general, not specific to youth versus family engagement, found engagement important and valuable in terms of early engagement, contributions to the project and efficient decision making. Some team members highlighted the value engagement in initial project development:

I think for youth and families that sit there with the people designing the project right from the beginning, it is an amazing idea. It’s so critical. Because often, families get thrown in halfway through or at the end and it’s kind of just like ‘oh how do you think this is?’ and ‘if you like this?’ and it’s way too late then. So, they were actually part of the design process, which I think was fantastic. (Participant A)

Team members further expressed how experiencing early engagement in research influenced their approach to research:

Having the community representation and youth and family there really has been the biggest […] but the best experience for me – and seeing really how we can integrate from the very beginning the youth and families and community into these large academic, scholarly research studies. It re‐shaped how I will do research, like, forever moving forward. (Participant B)

Youth and family members made important contributions to the YouthCan IMPACT project. Youth and family were seen to inform various services, ideas and outcomes throughout the process. In some cases, team members noted that engagement fostered more efficient decision‐making processes:

Before starting this study, I thought it was going to take more time to make decisions having youth and family engage and clinically; when I have done that for research it has taken longer. But really they made our decisions quicker because we would really quickly say ‘no, you could not use this because of A, B and C’. We would all sit around the table and go ‘Okay, we didn’t even think about that’. (Participant B)

#### 3.1.2 Engagement‐promoting environment

Many team members recounted features of the engagement environment. They noted that prior to YouthCan IMPACT, there was a lack of inclusion of youth and family voices in projects, and this changed with the YouthCan IMPACT project. One team member recalled how engagement was first put into place, ‘from my understanding is, everybody wanted [engagement]. But nobody did it, so we did it’. (Participant D).

Some team members cited inclusivity; they found that it was a non‐judgemental, safe, friendly and youth‐friendly space. A few participants remarked on youth and family‐friendly language as an essential component of engagement that fostered a safe space. Notably, the presence of youth and family in the research setting was seen to create a safer space for better discussions among all team members:

I think in part we have youth and families in the room, and I think that also makes people more attentive to like ‘this has got to be a space where it’s okay to say stuff’ and where people will not be made to feel bad because they said something that disagrees with someone else, or is maybe not well thought through – like, they say it out loud, and the minute they say it, they want to take it back. […] We’ve agreed implicitly to operate in a way that facilitates that sort of open discussion and transparency. (Participant E)

#### 3.1.3 Engagement as a complex, but game‐changing process

Many team members cited engagement as a complex and iterative process, with regard to the complexity of navigating differing voices in the co‐creation process, especially when youth voices differ from family voices. However, they saw this as a game‐changing and positive feature of engagement. Team members further discussed their experience with the YouthCan IMPACT project as an iterative process, involving extensive consultation with the youth and family team, i.e., co‐creation. Some team members expressed that they learned from the youth and family involved.

‘[Engagement] was such a great innovation, and something that I think the community [group] learnt from. And we’re looking at ways to do that in our organization for a lot of things. So that was certainly an enhancement to the community [group]’ (Participant A).

#### 3.1.4 Building strong team relationships with youth and family members

Team members cited building strong relationships with the youth and family members. Some had prior relationships with the youth and family members—this was seen to facilitate the research process: ‘I think the existing working relationships that we had from our previous opportunity to submit grants together was really helpful’. (Participant F). A few participants reflected on youth and family members being nervous at first. This was seen as temporary, where youth and family members quickly opened up as team relationships developed:

I did think in the beginning […] the families and youth were a little bit nervous about ‘Am I being heard? Am I a part of this?’ […] But as relationships developed, I think there was more of a balance with that. (Participant G)

Transparency, honesty and trust were also reported contributing to the positive relationships. One team member commented on the progressive growth of their relationship with the youth and family engaged:

There were a few lunches that we did together as a team, which I think broke the ice; which was great taking out sort of the boardroom or the office sort of community to a restaurant or just having lunch. Just getting to know each other. […] I think relationships are key, and that allows you to be more honest, which has been our model. (Participant G)

Overall, research and community team members expressed interest in working with youth and family members as a valuable part of the team. Team members further reported good relationships with youth and family team members, and perseverance within the team. These were seen as facilitators to a strong engagement process, fostering a positive impact on the project.

#### 3.1.5 Barriers to engagement

Many team members identified structural barriers to engagement. For one, the newness of engagement at the institution was seen as a significant barrier. The team expressed being among the forerunners in the field to rigorously incorporate youth and family engagement into mental health research and service design to this extent locally and in research as a whole. One team member reflected on the overall research environment:

The industry, if I can call that, is so far behind where it needs to be in terms of adequately meeting the needs or addressing the needs of youth and family members. There are still huge gaps. And I feel like, if I were the youth of a family member sitting around the table, I might still feel disappointed – even though from where I sit as the person that, one of the principal investigators, I know that this has been way more robust involvement than any other research project that I’ve been involved with. (Participant E)

Money and time, often paralleling each other, were seen as prominent barriers. This included funding limits (*e.g.,* no funding for a youth coordinator in earlier stages), tight timelines set by funders and limited time for meetings. Interpersonal barriers, such as differences in opinions among diverse team members, made it challenging to compromise between all of the voices. One team member pointed out the complexities in compromising as a barrier:

[Youth and families] often don’t have the same opinions, so that’s complicated things. It’s like trying to decide whose opinion you should be listening to, and so it was always a compromise between what’s feasible, what’s the methods group for, what we thought was as good study, and then what the youth and families thought was an acceptable intervention and an acceptable method. (Participant F)

Lastly, one team member expressed a busy schedule as a barrier:

There is just so much going on. It’s interesting just to see how many different things can happen at once. And that’s not necessarily a good thing, it’s just incredibly busy. (Participant H)

Overall, general feedback from team members supported the importance of youth and family engagement while highlighting both facilitators and barriers to successful engagement.

### 3.2 Findings specific to the engagement of youth

#### 3.2.1 Youth engagement is valuable

Similar to discussions around engagement in general, all team members who discussed youth engagement in particular found value in the youth contributions to the project and the impact of youth engagement on researchers. Almost all team members reported youth engagement as improving the project. Team members found that youth provide various levels of feedback and active participation, which was valued. One team member commented that ‘actually having youth to voice it when we're making those decisions, as opposed to saying “this is what we heard from youth in our reports” has been really, really helpful. I think their input has been invaluable’. (Participant G).

Youth were also seen as having a positive impact on team members. One team member expressed the impact youth had on their research career:

Having the youth involved and their input into everything has been a fantastic experience for me. (…) And their participation has made the project 10 times better. So, you know that whole experience for me, has been one of the most important experiences in my entire research career. (Participant I)

#### 3.2.2 Environment for youth engagement

Similar to the general findings, team members observed a learning curve for youth new to the team in the earlier project stages. They also noted that youth provided less feedback at the beginning, compared with later stages. However, they noted this was temporary, as youth soon felt comfortable speaking up. One youth team member recalled facilitators of the youth engagement process, such as pre‐briefs and de‐briefs for meetings:

As we got more comfortable and got to know things better, more actively participating. Then, before those meetings, having a pre‐brief with [the Project Coordinator] to talk […] and having that time to go over any terms or like any procedures that we weren’t that familiar with. And then also a debrief at the end if there is anything else to follow‐up on, or if there is any feedback that we didn’t want to share in front of the larger group. (Participant J)

A few team members observed youth being passionate and engaged, where one participant described youth as ‘interested, committed, and so when they reached out to other youth, they could be really genuinely engaging’ (Participant G). Overall, team members, including youth, were seen to be treated as equals. Team members also reported diversity among youth voices. One team member reflected on the Youth Advisory Group recruitment: ‘we wanted to […] have broad representation: so, youth who, you know, maybe, are naïve to the specific services, to think what would they like. But also, youth who are engaged with the services to have some voice’. (Participant G). A few team members commented that the Youth Advisory Group made it possible to include younger youth, which was a practical piece of the environment.

#### 3.2.3 Barriers to youth engagement

Team members identified barriers in the earlier stages of youth engagement, such as logistical barriers, lack of continuity, the need for stronger supports in earlier stages and resistance to change. Logistical barriers included scheduling and time management. Other barriers include a lack of continuity in the youth engaged (*e.g*. new youth are not fully aware of the inputs provided by previous youth), youth growing out of their roles and a lack of youth without research or service development experience.

Some team members discussed the need for stronger supports early in the project. One participant talked about the need for a youth coordinator in the earliest stages. Another participant reported that having the Youth Advisory Group earlier on would have been beneficial:

If it had happened way sooner, it would have been way more effective than it is now. Because again, you’re getting into like – just like check with them, see how they feel, basically just like dip your toe in the youth. […] the youth have brought up points that we were like ‘well we can’t change this now’. (Participant D)

This reflects challenges with regard to continuity, as youth were engaged in the very early stages of the project. However, not all team members were fully aware of earlier engagement and may have experienced different opinions with prior youth engaged in project. Lastly, one participant reported resistance to certain changes when decisions have already been made, reflecting similar sentiments team members expressed regarding differences in opinions in the general category. For instance, the participant discussed being asked about the new wall colour of one of the clinics, on which other youth had previously been consulted, but not being able to change it. This showed how different youth may have differing opinions and establishing agreement can be challenging.

#### 3.2.4 Improvements to youth engagement

Team members reported room for improvement in youth engagement, expressing that engagement is an iterative process. They highlighted that a smoother transition was needed for new youth entering their roles. One team member suggested, ‘if you know somebody else is going to take over, maybe put them in a few meetings. Just to, yeah, get the feel for it, warm them up to it’ (Participant D). Another suggested more effective communication between youth and other team members, stating that emails were ineffective.

Team members suggested furthering the reach of recruitment to include youth with no experience in research and service design. One team member suggested:

I think when you pull youth who have a lot of experience in the field, and [acting] in this role and interacting with their agencies, you don’t necessarily get a clear view of what the general population who largely hasn’t had these experiences would actually feel. So I think there could have been a little bit of effort made to reach outside of, kind of, the usual suspects to find a little bit more of a unique voice or, kind of, an unbiased voice. (Participant J)

Lastly, one team member mentioned how the Youth Advisory Group could have been used more for feedback. This team member suggested, ‘thinking a little bit more critically about which points in time we were going to need the most youth voice, and having those meetings set out ahead of time, and not so ad‐hoc’. (Participant J).

### 3.3 Findings specific to the engagement of family members

#### 3.3.1 Benefits of family engagement

Team members expressed the importance and value of family engagement. Mirroring sentiments regarding youth engagement, team members found the family voice crucial to the YouthCan IMPACT project. Family members made several contributions to the service delivery pathway and improvements to the service model by integrating an intervention for family members. Team members reported good family engagement, including the initial project proposal development process:

[Family] has been so consistently engaged in [providing their] input […] I think that’s really valuable for us. Really making sure and keeping alive. Again, you could say it’s in the literature, but [their] voice is there in a strong way, saying, you know, ‘I’m telling you to have things – you need to engage parents, have things for parents and you need to do things in a very evidence‐based consistent way with fidelity’. I think those have been huge contributions (Participant G)

Family engagement was also seen as rewarding:

I would say enlightening. For me, I would say an amazing learning experience. I’ve been growing. (…) To be able to contribute for a project is so rewarding and so incredible. (Participant K)

#### 3.3.2 Barriers to family engagement

Similar to team members’ sentiments regarding barriers to engagement in general, participants reported family engagement being a rushed process. One participant felt family members were less supported than youth, where there was a stronger focus:

…if you look at the Division that is working on the project, it is the family, child, and youth unit. Family members tend to be forgotten often […] But, yeah maybe we’re doing more with youth. Maybe we should be doing more with family members as well. (Participant K)

#### 3.3.3 Improvements to family engagement

Although team members were appreciative of the family engagement process, some identified room for improvement. Most importantly, team members stressed the importance of enhancing family engagement:

…there is always space to make more room for the family engagement aspect of it. I know the emphasis is lot on the youth, and the family sometimes tend to be forgotten. So, I’m very happy that we have a place of voice at the core team to make sure that we don’t forget families. I find that in the recovery process, it’s ‘together as one’ that we get the best outcome, right? (Participant K)

## 4 DISCUSSION

### 4.1 Summary

This study explored the experiences of YouthCan IMPACT team members with youth and family engagement in the design and development of integrated services and a related research project. Participants strongly endorsed the value of youth and family engagement. We draw on the results of our interviews to illustrate general, youth‐specific and family‐specific engagement experiences. Broadly, these categories encompass team members’ overall experiences with youth and family engagement, including the value and benefit of engagement, features of the environment, team relationships, barriers and potential areas for improvement. These novel findings provide a nuanced understanding of the experiences of team members during the intervention and research project development process.

The McCain Model of Youth Engagement was applied to ensure engagement throughout the project process,^7^ where substantial levels of engagement were seen. Our study suggested the program achieved authentic youth and family engagement consistent with the principles of the McCain Model, whereby youth and family members were: (a) recognized as equals by team members; (b) involved in various aspects throughout the research process; and (c) engaged in shared decision making.

### 4.2 Comparison with existing literature

The work environment plays a critical role in fostering trusting and collaborative relationships among inter‐professional teams.^34^ Honesty and trust were seen as key to building team relationships in this study, as well as earlier studies on youth engagement,^35^ family engagement^3^ and service user engagement.^27^, ^36^ Although prior research reported mistrust as a barrier,^27^ participants in our study focussed on trust as a strength. Several other features of the environment facilitated engagement, including non‐judgemental, safe, youth‐friendly spaces, in which youth and family‐friendly language was used. These features are reflected in several studies on key attributes of youth engagement.^4^, ^16^ A broad range of youth voices were incorporated into the project through expansive engagement, enabling the team to draw on different perspectives as highlighted in the McCain Model of Youth Engagement.^7^

The strong team relationships with youth and family members were key to facilitating successful engagement. This created psychologically safer spaces for discussions, reflecting a study by Halsall et al,^35^ which highlighted positive relationships between youth and staff as key to youth engagement. Integrated knowledge translation research shows that building on previous relationships with service users can lead to positive research outcomes.^37^ Indeed, team members suggested pre‐existing relationships with youth and family members facilitated the research process. Lastly, engagement as early as possible supported success, as identified in previous studies on principles and best practices in service user engagement.^38^, ^39^

Several studies have outlined potential barriers to youth engagement, such as limited practical resources and funding.^40^, ^41^ Despite the value of engagement, participants also highlighted barriers, such as time and funding constraints. These findings align with a study by Harrison et al^39^ and a systematic review by Domecq et al^13^ on service user engagement in research, where time constraints (*e.g.,* more time needed for research) were seen as major barriers to engagement. Time, such as lacking time to support youth engagement, was also reported as a barrier in a study by Hawke et al.^18^ Barriers to family engagement in the current study parallel previous findings^42^ that caregivers experience limited involvement and exclusion from their youth's care. Youth turnover has been considered a structural barrier to engaging youth^43^; this parallels the present study, which identified youth ‘growing out’ of their roles as a barrier. Youth team members also mentioned experiencing team resistance to changes when decisions had already been made, highlighting the importance of clear and transparent communication.

It is important to note that the YouthCan IMPACT project has evolved and addressed many of the challenges since the start‐up phase. For example, a youth engagement coordinator has joined the team, together with multiple youth staff members and broader youth engagement in the McCain Centre as a whole, rather than specific to the project. This has expanded the scope of youth engagement and potentially addressed multiple barriers identified, such as time management and scheduling barriers, and a need for stronger supports in early stages. However, as this is beyond the scope of the current study, a follow‐up evaluation would further clarify the degree by which the barriers have changed since the start‐up phase.

Few studies have examined the perceptions of engagement by broad project teams, particularly relating to youth mental health. The powerful and impactful nature of youth and family engagement has been found previously.^3^, ^11^, ^13^, ^44^ Happell et al,^11^ for instance, found that engagement brought value and improvement to mental health research. Our study furthers these results by revealing that consistent and authentic youth and family engagement is achievable and improves a project. There is a paucity of research on family member engagement in youth mental health research and service development; one previous study discussed the value of family engagement in obesity prevention programs.^3^ Our study demonstrates positive impacts of family team members, showing promise for effective family engagement.

#### 4.2.1 Implications for practice and research

This study demonstrates that youth and family members can be engaged in service development and a randomized controlled trial project, where the engagement process is perceived as valuable to the project team. Indeed, the engagement process had numerous valuable impacts on the project. For example, it was through youth engagement that the primary outcome measure was selected, research staff were trained to work with youth and family members effectively, the project website was developed and a family‐focused intervention was added to the intervention pathway.^9^ These, among other contributions, were key to shaping the project and the intervention, and highlight the value that engagement imparted upon it.

Engagement is increasingly valued by research organizations, such as the Strategy for Patient‐Oriented Research at the Canadian Institute for Health Research in Canada ^45^ and the Patient‐Centered Outcome Research Institute in the United States.^46^ It is important to increase researchers’ capacity for youth and family engagement, through capacity development initiatives such as the INNOVATE Research project.^18^, ^40^ However, future work is needed to enhance and scale youth and family engagement practices. Informed by the facilitators and barriers to engagement, recommendations for future projects aiming to incorporate engagement are shown in Box 1.

Box 1 **Recommendations for future projects incorporating youth and family engagement.**

1. Involve youth and family members as early as possible, ideally at the project design stage.
2. Ensure a broad range of youth voices are included to accurately reflect service user needs (*e.g,* youth who are younger and have no experience in the field). Plan for flexibility in evolving youth roles as their interests may shift and they may no longer represent the target population, over time.
3. Promote safe and friendly environments as per models of engagement.^7^, ^16^ Emphasis should be placed on relationship‐building and having appropriate supports in place.
4. Engage in difficult discussions among researchers youth, and family members, as these may result in promising outcomes.
5. Family members can provide important insight into youth services; thus, incorporate family engagement alongside youth engagement.
6. Be cognizant of the structural barriers to engagement, such as limited institutional buy‐in to engagement practices. Consider strengthening structures to support engagement and address barriers.
7. Consider capacity development initiatives to increase engagement skills and buy‐in among researchers and institutions.

#### 4.2.2 Limitations

This study has several limitations. The study was limited to team members on the YouthCan IMPACT project; therefore, transferability is limited. The small sample size, especially of youth and family participants, is another limitation. Additionally, as this study was a secondary analysis of existing data initially collected to understand the start‐up process of the project as a whole, it was not directly designed to evaluate the benefits and barriers to youth and family engagement, which limits the study to an exploratory design.^47^ However, given the paucity of research on the impacts and experience of youth and family engagement in research and service design from diverse perspectives, this study provides a rich description of a relatively uncharted area. This study is limited to the start‐up phase of the YouthCan IMPACT project; as the team has learned from their experience, engagement has evolved, and improvements have been made.

## 5 CONCLUSION

Youth and family engagement are a valuable and impactful process that plays a critical role in research and clinical service pathway design. This study demonstrates the positive experience of youth and family engagement in the YouthCan IMPACT project, while revealing potential barriers teams may face. When teams make the sustained effort to implement engagement, research and service design is improved. Future research should evaluate the outcomes of youth and family engagement, such as the long‐term impact of early engagement in research and service design, and further focus on family engagement.

## 6 DATA SHARING STATEMENT

The data that support the findings of this study are available from the corresponding author upon reasonable request.

## CONFLICT OF INTEREST

The authors declare no conflicts of interest.

## AUTHOR CONTRIBUTIONS

Natasha Sheikhan contributed to the analysis, interpretation of data, drafting of the manuscript and revision of the manuscript. Lisa Hawke contributed to the acquisition of data, analysis, interpretation of data, revision of the manuscript and final approval of the version to be published. Kristin Cleverley contributed to the conception, design, revision of the manuscript and final approval of the version to be published. Karleigh Darnay contributed to the analysis, interpretation of data, revision of the manuscript and final approval of the version to be published. Lynn Courey contributed to the design, interpretation of data and final approval of the version to be published. Peter Szatmari contributed to the conception, design, revision of the manuscript and final approval of the version to be published. Amy Cheung contributed to the conceptualization, design and operationalization of the study, interpretation of the data and editing of the manuscript. Joanna Henderson contributed to the conception, design, revision of the manuscript and the final approval of version to be published.
